# The use of instructional videos to compensate for flexible physiology learning during the pandemic of COVID 19

**DOI:** 10.1186/s12909-023-04924-8

**Published:** 2024-01-10

**Authors:** Noha N. Lasheen, Maram M. Fawzy, Mostafa B. Ibrahim

**Affiliations:** 1 Associate Professor of Medical Physiology, Faculty of Medicine, Galala University, Suez, Egypt; 2 Associate Professor of Medical Physiology, Faculty of Medicine, Ain Shams University, Cairo, Egypt; 3 Undergraduate Students, Medicine and Surgery Program, Field of Medicine, Galala University, Suez, Egypt

**Keywords:** Student-centered learning, Instructional videos, Physiology teaching

## Abstract

**Background:**

This study aimed at using instructional videos in physiology created by students to improve the process of learning Physiology especially during the COVID-19 Pandemic which enforced the lectures to be online. Additionally, it allowed students to visualize and understand clinical scenarios and the physiological reasons behind them while assessing how much they stand to gain from the experience.

**Methods:**

This study is a project to implement FAIMER, ASU MENA-FRI Institute, Cairo, Egypt. In a foundation course for first-year medical students, the instructor utilized a variety of instructional methods including lecture, small group discussion, individual assignments, and reflection. Students were randomly allocated into 18 groups, then a topic in their physiology curriculum was chosen and they formulated a related case scenario, thereafter a video was made by themselves. This intervention was rewarded by activity mark in their course. Post-project questionnaire was used, and an external reviewer evaluated the videos presented by students. This study obtained IRB approval from the Faculty of Medicine, Ain Shams Medical Ethics committee.

**Results:**

the project helped students to improve their skills in problem-solving, teamwork, active learning, communication, planning, and time management. In addition, it also increased their confidence in their abilities to learn, face unexpected challenges, and achieve goals, while considering new life opportunities, those which became an option when the students searched by themselves and learned more about the different angles of medicine.

**Conclusion:**

Compared to the traditional lecture format that focuses on memorizing definitions and theoretical structures, instructional videos can be regarded as an innovative teaching tool and a unique medical education method that allowed students to participate more in the learning process even if their lectures were online. This article proposes an active learning method in undergraduate medical education which compensate for limited face-to-face attended during the pandemic.

## New and noteworthy

This study has applied an effective tool for interactive learning, which was instructional videos made by students and were disseminated among all students in the same level and to students of the next academic year to ensure student-centered physiology learning and to encourage more students to create and implement their own stories, thereby realizing short outcomes of use of technology in physiology learning.

## Background

Because the COVID-19 pandemic has forced colleges and universities to use a large amount of educational content online in a short time frame, online learning followed resulting in the enhancement of the requirement of instructional videos to reach learners at a distance. Instructional videos were used to help more students learn in remote settings such as during the pandemic [1].

Some instructors preferred to include educational slides behind them [2] and/or use a board to allow the instructor to write notes [3]. These trials to incorporate the instructors themselves in the video, may not be considered an effective instructional design technique because the research in the area has not been synthesized [1].

During such hard times worldwide, online teaching caused learners to be physically isolated from instructors and fellow learners [1]. Therefore, it is essential to incorporate the knowledge with technological methods to elucidate an effective learning process.

Traditional lecture has proven advantageous in being informative and providing a sense of confidence to students regarding the accuracy of the taught material [4]. Nevertheless, it lacked the necessary interaction, reflection, and collaboration in the classroom [5]. It was found that videos have a positive impact on education when integrated with traditional classes. Thus, modern educational approach of instruction was recently used to get more benefit than using traditional education alone [6].

In a rapidly developing health care system, developing skills such as critical thinking and decision-making are crucial. The use of routinely used techniques such as memorizing and mnemonic schemes without marking conceptual relationships does not create a database for future references [7]. They only give the students the chance to retain information long enough to pass an exam. On the other hand, integrating multimedia tools in the learning process has provided a more beneficial technique. This method not only clarifies conceptual relationships and stimulates students’ interest in learning, but also enables teachers to assess a student’s learning status [8, 9].

In 2017, a study regarding the relationship between health care students’ use of technology and their achievements in a Physiology course at the University of Dammam was published. The study reported a significant association between the use of technology and the rate of achievement in health colleges. It also observed an increase in students’ reliance on technology for their academic needs. This further proves the importance of incorporating different technological methods as learning tools. One of these tools is using videos to present a case in a certain topic [10].

Student-centered teaching aims to create an environment that encourages active learning, reflective and critical thinking, and collaborative problem-solving [4]. The further addition of instructional videos to lectures can turn a teacher-centered classroom into a student-centered classroom [5].

Up to the authors’ knowledge, there is a limited use of instructional videos in medical education [11]. Therefore, it is of value to introduce such a promising tool in a newly established university in Egypt *(Galala University)* especially during the limited face-to-face education during the quarantine.

## Methods

This study aimed at introducing instructional videos created by students to compensate for unavoidable online education. This not only helps to spread medical knowledge, but also helps to acquire new skills and abilities.

This study is a project to fulfil FAIMER, ASU MENA-FRI Institute, Cairo, Egypt. In a foundation course for first-year medical students, the instructor utilized a variety of instructional methods including lectures, small group discussions, individual assignments, and reflections; this utilization of materials served as the intervention. The learning objectives of the intervention were to enhance the students’ understanding of physiology topics and strengthen their communication and presentation skills through the creation of instructional videos, this was theoretically based on principles of active learning and student-centered teaching approaches, which have been shown to improve engagement and knowledge retention. It is crucial to note that the instructor had an experience with prior use of an instructional video project, (Medical Workshop in IUPS, August 2017, Rio, Brazil).

This study obtained IRB approval from the Faculty of Medicine, Ain Shams Medical Ethics committee.

### Participants

The participants eligible in this study were first-year medical students enrolled in the Faculty of Medicine at Galala University. A total of 139 students were included in the study and randomly allocated into 18 groups, with n = 6–7 members in each group. Each group was asked to make an instructional video using case scenarios with the help of freely downloaded applications as material, for example tools for video editing, voiceover recording, and adding animations. The goal was to produce a short video (5 to 20 min in duration) using voiceover to explain a physiology topic in their foundation year. The sample size of 139 first-year medical students was determined based on the available student population in the Faculty of Medicine at Galala University. Random allocation of students into groups was performed to ensure equal distribution and avoid bias.

### Procedure

In each group, a leader was chosen by their teammates, and they were free to choose any topic from their physiology curriculum. The instructor assigned the project in the first week of the Fall 2020 semester. They started working on their project to be completed two weeks before the final exams. Ten marks (out of 150) were assigned to such student activity. The instructor monitored their progress and supervised the cases created by them throughout the semester. The instructor required one meeting with each group before the final project deadline (online or physical meeting).

For interaction, Students were encouraged to exchange email addresses, phone numbers, and communicate in person with their teammates from day one. Students were allowed to work on class days on their projects, particularly to meet with their group members, discuss project ideas, or edit their final videos. And due to the coincident COVID 19 Pandemic in Fall 2020, online meetings were also used to exchange ideas and complete the project according to the time frame.

After the delivery of 18 digital case scenarios, a zoom meeting was used to present the projects to other groups. All cases were available for first-year medical students to be used as supplementary materials to their curriculum for this batch. In addition, these supplementary materials were used by the instructor to the following two batches in Medicine and Surgery Program, Galala University, they were asked to do a reflection on each video they watched as a part of their portfolios.

In first batch of Fall 2020, the first group presented a spectacular digital case scenario one month before the deadline. The project was inclusive of the discussed topic, clearly narrated, and included animations that made it easier to understand the discussed topic. The instructor uploaded the first instructional video to all students to encourage others and to get more benefit from the design, implementation, and presentation. The instructor also gave two students of the first group the opportunity to assist on this research paper (M. M. F and M. B. I) as an incentive and a reward.

A satisfaction survey was disseminated among students of the first batch to collect their impressions, strengths, and comments on the performed task. Additionally, an external evaluator was volunteered to evaluate all instructional videos according to a rubric, showed in Table 1.

**Table 1:** Rubrics for Instructional videos

	5	4	3	2	1	0
Point of view						
Content						
Resources						
Curriculum alignment						
Organization						
Student cooperation						
Camera and images						
Titles and credits						
Sound						
Language						
Pacing and narrative						
Transitions and effects						

### Grading rubric

The instructor created a grading rubric to evaluate each group’s final projects (Table 1). A volunteer reviewer received instructional videos made by students to validate them and his comments are displayed in the results section.

### Statistical analysis

Data were collected, revised then subjected to statistical analysis using one-way ANOVA performed by SPSS.21 program (IBM Inc. Chicago, Illinois, USA).

## Results

First, the instructor observed the students’ knowledge and understanding of homeostasis, biophysics, membrane potential, nerve impulse, and hemodynamics presented in their instructional videos. Students showed an in-depth understanding of these concepts.

Second, using a case scenario helped students in gaining more information about the clinical application of the course in health promotion practice through watching their final instructional video projects. Students demonstrated creativity and innovation in their videos. Some of them made integrated cases (histology and clinical practice, such as history taking, which were used to present the physiology topic as well).

The following results were obtained from a student satisfaction survey, in which each student expressed their opinions regarding a multitude of areas related to their projects.

**Fig. 1:**
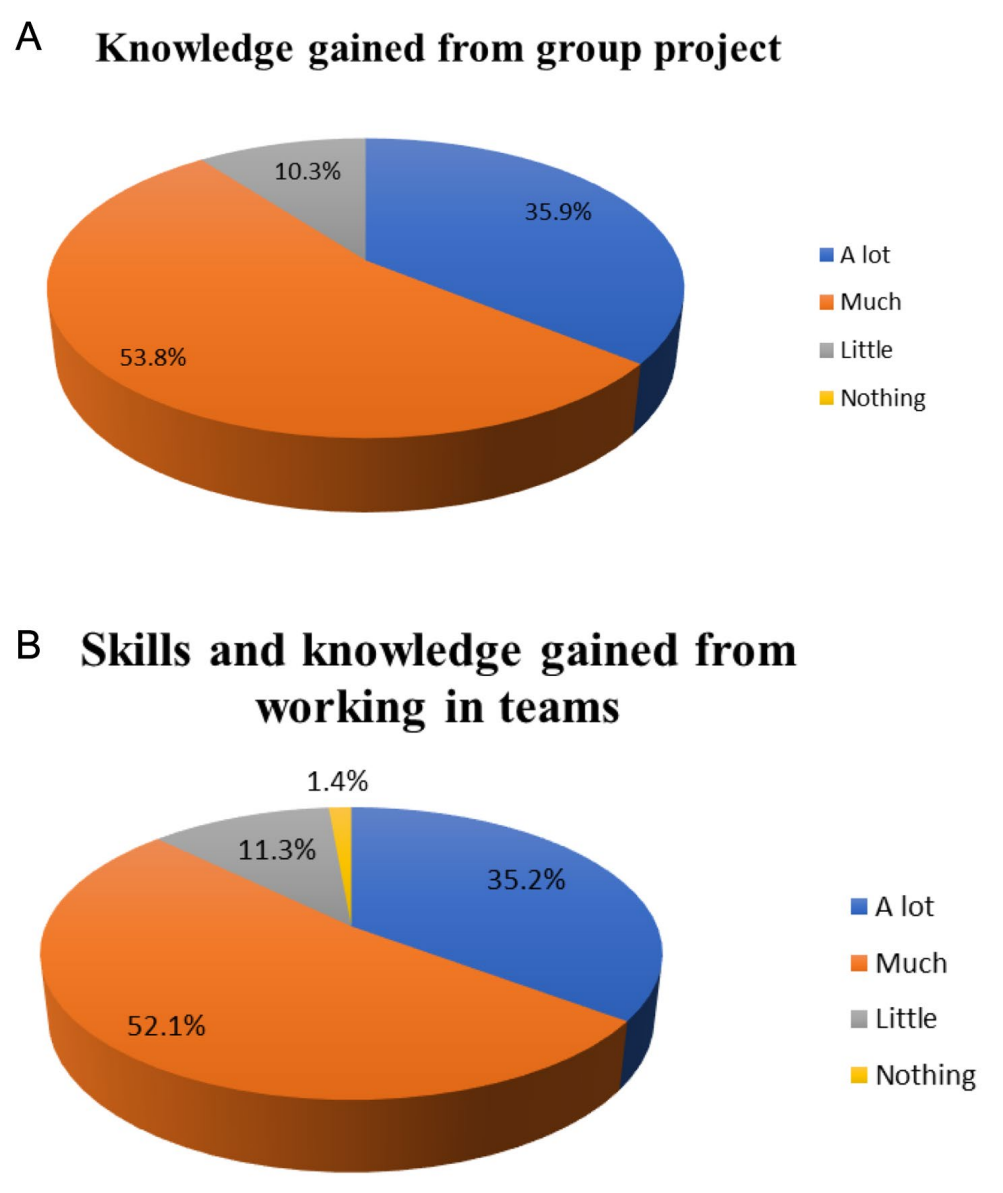
(**A**) Students’ responses analysis about knowledge gained from the project. (**B**) Students’ responses analysis about knowledge and skills gained from the project

Regarding the extent of knowledge gained by the questioned medical students, the question referred to the general experience provided by the group project. The choices included were a lot, much, little, and nothing, as shown in Fig. 1A and Table 2. It is clear from the pie chart that more than 85% of students reported gaining either a lot of or much knowledge from the group project experience, while a minority reported gaining little knowledge from the group project experience.

**Table 2:** Student responses on the satisfaction survey

	Strongly Disagree	Disagree	Neutral	Agree	Strongly Agree
**My project developed my problem-solving skills.**	0%	0%	10.3%	53.8%	35.9%
**My project helped me develop my ability to work as a team member.**	1.3%	1.3%	11.7%	35.1%	50.6%
**My project helped me to be an active learner.**	0%	0%	6.5%	37.7%	55.8%
**My project has improved communication skills (oral, verbal, and written).**	0%	0%	18.2%	44.2%	37.7%
**My project helped me develop the ability to plan my work.**	0%	0%	6.5%	39%	54.5%
**My project helped me properly manage time.**	0%	6.5%	14.3%	41.6%	37.7%
**As a result of my project, I feel more confident about tackling unfamiliar problems.**	0%	2.6%	21.1%	46.1%	30.3%
**My project has made me more confident about my ability to learn.**	0%	1.3%	6.5%	49.4%	42.9%
**As a result of my project, I am confident about achieving my goals.**	0%	0%	10.4%	51.9%	37.7%
**My project has helped me think about new opportunities in life.**	1.3%	1.3%	35.1%	40.3%	22.1%
**Overall, I was satisfied with the quality of my project.**	0%	2.6%	9.2%	39.5%	48.7%

However, Fig. 1B represents the survey’s findings regarding the skills and knowledge gained from working in a team. The choices were the same as used before: a lot, much, little, and nothing. This pie chart demonstrated that more than 87% of the questioned students reported gaining either a lot of skills or much skill and knowledge from the teamwork aspect of the project, meanwhile, a minority reported gaining either little or no skills and knowledge from the teamwork experience.

**Fig. 2:**
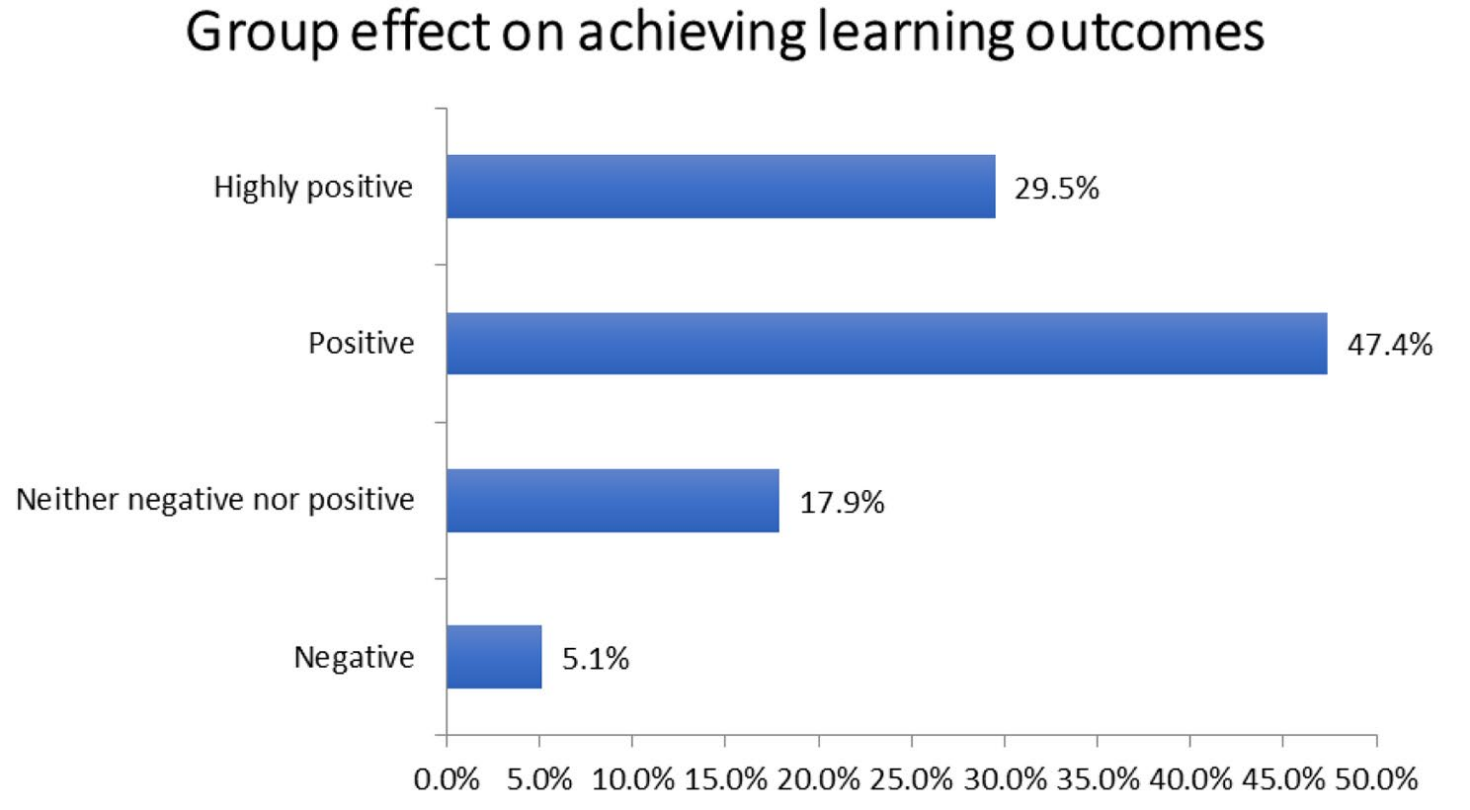
Analysis of students’ responses about group effect on achieving learning outcomes

Figure 2 represents how the students thought the group increased their ability to achieve their learning outcomes. The choices included highly positive, positive, neither negative nor positive, and negative. The results illustrated in the bar graph displayed positive effects in more than 76% of students.

Table 2 shows the students’ general reflections on the abilities they acquired after finishing their projects regarding development of problem-solving, team-working, active learning, communication, planning, and time management skills, as well as increasing their confidence regarding tackling unfamiliar problems, their ability to learn, achieving their goals and thinking about new opportunities in life. The possible answers were: strongly disagree, disagree, neutral, agree, and strongly agree.

More than 88% of the participants reported their agreement with that this project helped them develop their problem-solving skills, while 10.3% were neutral regarding the matter. However, no students disagreed.

Regarding developing team-working skills, also a considerable percent agreed, however, less percent showed either neutral or disagreement in this point. Similarly, more than 80% of all participants reported their agreement with improvement of active learning abilities. Around 75%of students mentioned that they gained an improvement of the students’ oral, verbal, and written communication skills. Also, more than 90% reported that they got much benefit from planning their work independently.

Regarding time management skills, 78% of students agreed with the gain of time management skills they acquired, without any student strongly disagreeing.

The results also show that the majority of students reported an increased sense of confidence regarding their ability to learn after being involved in this project, also, it helped them acquire confidence regarding tackling new and unfamiliar problems.

Regarding the confidence factor about achieving the goals, 88% of the students agreed with that this project made them confident about achieving their own goals without any of the disagreeing whatsoever. About life opportunities, around 60% agreed that this project helped them think about new opportunities in their lives.

Concerning the overall satisfaction with project quality, more than 88% of the students agreed on finding the quality of their projects satisfactory.

The survey included optional review questions as well: “What is the most impressive point or part that got your attention?” and “Any suggestions for the improvement of the idea?”

As shown in Table 3, regarding the question about the most impressive part, many students reported that it was the gained skills of collaboration and time management. One of the students said: “It was mostly how well the team worked together and helped each other. Every team member stepped up when needed and helped as much as he/she could, even when their specific tasks were done.” Some reported that it was the “challenge of active learning” that was most impressive, about which a student remarked that “Although I was researching and writing about a mechanism that I only found significant in the human body, its clinical applications changed the way I started approaching new knowledge in medicine and seeing its importance even if it is as simple as water moving from one side to another.” Others reported that the most impressive part was the way each team presented their final projects, where one student said that “It was compelling (more than expected) to watch other teams’ projects because each team had their unique touch/twist on the idea and displayed overall the lessons in a very intriguing manner. I enjoyed learning from the students themselves for a change. Students generally think the same way so they could convey the ideas in a way that is closer to a student’s understanding.”

**Table 3:** Student responses on the satisfaction survey “What is the most impressive point or part that got your attention?” and “Any suggestions for the improvement of the idea?”

ID	What is the most impressive point or part that got your attention?	Any suggestions for the improvement of the idea?
1	My team’s ability to work under pressure	N/A
2	Competition between teas to have the best projects which made all of us do his best	N/A
3	After project I started online video editing course to help me in the upcoming projects	N/A
4	Making the PPT into a video, which (we) couldn’t do in time, but (we) did find a solution.	Perhaps tasks such as these could be done in pairs or trios. Four people in my opinion is a lot, and other than that there’s nothing I have to add.
5	Our attempt to have the best project is very impressive point for me	N/A
6	when my group and I have meeting in the university gave us trust of each other and also give us confident to work together	I think if we dived each group project members according the skill level, each group will achieve the team work and make perfect project
7	Work as a team	No it was great
8	The team work and time management	No, it is good
9	The team work and time management	No, it was perfect
10	I was really compelled (more than expected) to watch other teams’ projects because each team had their own unique touch/twist on the idea and displayed overall the lessons in a very intriguing manner. I enjoyed learning from the students themselves for a change. Students generally think the same way so they were able to convey the ideas in a way that is closer to a student’s understanding.	N/A
11	Group members’ potential maybe…	N/A
12	Personally the challenge of actively learning to accomplish the task was the best part.	N/A
13	learning the medical application and how to make case scenario	not to make random selecting for group members and let them choose their mates because it makes too problems
14	That I have gained experience from my team members in dealing with problems and I learned from them new things	We have to increase such great works as I was impressed that I have participated in a such a useful work which will last for a long time
15	it is working in a team and our ambition to achieve the goal is the same and we must do it in the best way	yes, i have one that the time or the date that we will start to make the project should be a little bit of in free time not in stressful time like before the exams and the deadline is near and thank you for your interests
16	The most impressive point which got my attention was to make a case scenario.	I really have no suggestion to say and I want to thank our program director and our professor of Physiology Dr. Noha Lasheen who encouraged us and provided us with the important info to do projects like those. Thank you so much for everything.
17	The collaborative teamwork.	Nothing critical, but maybe making it more competitive would be interesting.
18	It was mostly how well the team worked together and helped each other. Every team member stepped up when needed and helped as much as he/she could, even when their specific tasks were done.	While I am very satisfied with my team, some other teams weren’t as lucky. My suggestion is that the teams would be more evenly distributed.
19	Working to fully appreciate the outcome of your work at the end no matter how better your colleagues did as long as you did your best, was happy for them and was grateful for all your efforts big or small.	More general engagement in the group and more assigned active roles to each member.
20	Team work were the most important thing	N/A
21	The lovely wishes for us from other colleges	N/A
22	The doctor’s sense of humor while teaching.	N/A
23	Positive competition between students	I can’t find any suggestions
24	Team work and improve my learning skills	N/A
25	How difficult it was, the project difficulty was underestimated	To make us present our project in different ways, as with using animation was a great idea, looking forward for new other ways
26	Work as a team	N/A
27	team work and trying new skills	N/A
28	Animations helped grab my attention and clarify concepts.	N/A
29	While presenting inside the university this feeling, which was a mixture of excitement and tension, was very wonderful	More organised team with smart leader
30	The ability to divide a project subject into tasks among the team but In the same time, The each individual in the project gain knowledge about the whole subject of the project	N/A
31	The most impressive point is how all the groups tried their best to make a competing project. And how all the groups tried their best to make a good project. I really didn’t expect that.	N/A
32	Learning new things	I think if we can choose our group the work will be better and easier
33	Team working was very smooth	N/A
34	Some of my colleagues work	N/A
35	Team work	N/A
36	The variety of the ways each teams presented the project	N/A
37		N/A
38	Me and my team’s improvement throughout the process of working on this project regarding the depth and quality of our collected data.	Maybe having one-to-one mentor with each team to proofread their scientific bases step by step.
39	Time management	N/A
40	A lot all the projects was nicely done all teams leaders and members worked hard	keep on encouraging people to work together
41	How much I can achieve and learn in 48 h	Making it more like TED talks where we present in a hall or an area surrounded by other students and focusing on our presenting skills rather than our animations
42	1- self learning abilities that we gained 2- successful team work experience	N/A
43	The communication between the group members and their availability and wellness to help	N/A
44	The motivation to finish the work.	N/A
45	Although I was researching and writing about a mechanism that I only found significant in the human body, its clinical applications changed the way I started approaching new knowledge in medicine and seeing its importance even if it is as simple as water moving from one side to another.	Allowing the students to choose there partners or at least have a say in it, will help students avoid uncomfortable situations.
46	Permanent follow up	N/A
47		N/A
48	The most impressive point is the importance of tonicity and team work with my friend.	I wish i could add more topics that i searched about and liked but me and my team made it more simple cause of limited time
49	working in a group and being able to solve problems that occur while working. Learning new skills from your colleagues.	I believe that it is a very good idea to let the students work together and prepare a wonderful project as it will make them very confident about their abilities and it will improve a lot of their skills. They may also discover new skills and qualities they didn’t even know that they have. It is a very effective way to learn as well.
50	I’ve had so much fun with my teammates creating scenarios and cases and discussing it and learnt alot from my teammates as in regards of editing skills and so	N/A

However, regarding the improvement suggestions, most students suggested a different distribution technique for the team members. One student suggested that “allowing the students to choose their partners, or at least have a say in it, will help students avoid uncomfortable situations.” Another student wrote: “I think if we divided each group project members according to the skill level, each group will achieve better teamwork and make a perfect project.” Nevertheless, the students had suggestions about the project’s limited timeline as well, including the suggestion that “the time or the date that we will start to make the project should be a little bit in the free time, not in a stressful time like before the exams.”

**Fig. 3:**
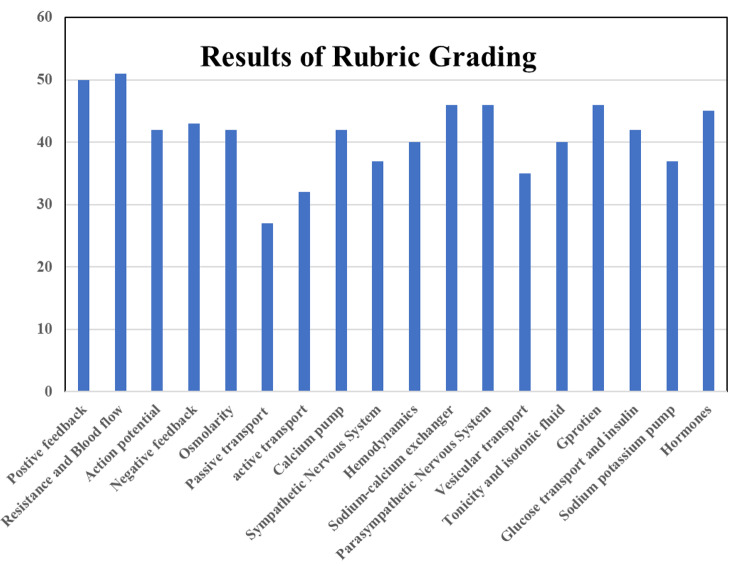
Results of grading rubric of all presented instructional videos

When analyzing the grading rubrics performed by the external evaluator, there was an average of 41 score out of sixty, as shown in Fig. 3. There was two instructional videos which gained more than 50 out of sixty, while eleven instructional videos got 40s. Four instructional videos scored 30s out of 60, only one video has a low score of 27.

### Short-term outcomes

They include active student participation, team working (students), mentoring by the teacher, increase the use of technology in the teaching/learning process by both teachers and students and increase teacher-student contact time. Also, another short-term outcome was realized which is sustainability, as the second batch were asked to do instructional videos in their second year in Cardiovascular module integrating physiology with other domains namely, pharmacology and pathology. This denotes that the first batch were considered as role models for the next batches and the student-centered learning were achieved.

## Discussion

This study revealed high percentage of student satisfaction about gain of knowledge, better time management, improvement of their oral, verbal, and written communication skills, enhanced confidence about achieving their own goals, higher sense of confidence regarding their ability to learn, development of their problem-solving skills and acquiring team working skills. All these aspects promoted good outcomes and helped in implementation of student-centered learning in such hard times during COVID-19 Pandemic.

Similarly, video production, combined with literature review writing was an efficient tool of learning. Most students found that video and literature review projects have emotionally positive impacts and they were motivated to work [12].

From the aforementioned results, medical students in first batch in Galala University have proved that use of technology, in the form of instructional videos, can be suited to validate better engagement in the learning process. In line, Beheshti et al. [6] mentioned that using videos in learning is a powerful educational method to improve learning results as well as learners’ satisfaction.

The value of forming instructional video can be suggested to realize better gain of knowledge and understanding the explained topic. In support, the process of developing a digital case scenario, especially as a group, was found to be very beneficial for medical students. These cases can help them identify key messages, summarize key concepts, and communicate to an audience. They also help build teamwork skills such as attentive listening, group participation, and a sense of being a part of an active unit [13].

In addition to gaining new information, developing an instructional video, and presenting it improves many skills such as organization, time management, communication, and presentation, which are only some examples of how implanting a digital learning tool can positively affect the educational process in a taught module, in this case, an introductory physiology module [7, 8]. In support, the evaluation made by the external evaluator herein, deduce in-depth knowledge and acceptable presentation skills in participants of this study.

On a related note, instructional videos promote the use of peer learning, as the students who created them are teaching a topic to their colleagues. In other words, it’s a form of student-centered learning (SCL). Some studies show that SCL can be used in physiology courses to help students learn [14–16]. It was suggested that the most complete and fruitful strategy for teaching physiology is providing knowledge in a manner that addresses multiple learning types. In other words, information should be given simultaneously in multisensory modalities [17].

Furthermore, the integration of different sensory inputs through combining various learning methods guarantees a higher rate of information delivery and retention [7, 17]. Additionally, when the students use their learning styles to develop their learning materials, the results are better than normal regarding the amount of information acquired from the course [18], denoted from presence of 11 instructional video with good quality, as measured by the grading rubrics.

On the other hand, peer learning helped in information gathering, designing, presentation, audio, voiceover, brainstorming, scriptwriting, and animating processes were evenly distributed among them. As the students received valuable personal feedbacks, this approach emphasized collaboration rather than competition [10, 19–21]. The results also complement the previous statements through stating how many students reported a positive effect on achieving their learning outcomes while working in groups (Fig. 2).

It is essential to mention some disadvantages of team working and peer learning. One of them is the improper distribution of work among students, resulting in unfair excess workload on certain group members. This sometimes leaves other group members feeling left out. Another one can be the spread of inaccurate information among students due to a lack of experience and knowledge. Fortunately, these disadvantages are minor drawbacks as they have simple solutions. As suggested by the students when asked for suggestions, properly distributing the workload, and letting the students choose their own group members might help solve this problem. Also, providing easy access to trustworthy knowledge sources and professors, and educating the students on the aspects of instructional videos are all efficient solutions regarding how to deal with these drawbacks [4].

Nevertheless, barriers such as lack of skill, poor communication, or inadequate task distribution can negatively impact the purpose of developing an instructional video. While technical skills can be improved through training, other like communication and inadequacy cannot. As mentioned above, the purpose of instructional videos is to convey information and important concepts in a manner that holds the viewer’s attention. However, these barriers defeat this purpose, either through not properly conveying the information or inhibiting a student’s willingness to participate in the process of instructional video development [22].

### Lessons learned

The instructor learned important lessons from implementing this class project. Active participation of students is essential and needs a planned teaching strategy, especially during the COVID19 pandemic.

A second lesson learned from this project was the importance of providing student feedback throughout the project. For many students, this was their first introduction to using digital media and technology in a class project. Students were allowed to create, edit and finalize the project before the last week of class. Also, groups were willing to share tips and advice with other groups in terms of creating and editing the final video, using the first presented project as a role model.

As first-year medical students, the students reported learning much from the experience. It allowed them to improve their researching skills and to enhance their team-working and communication skills. It also gave them the initiative to develop proper time management skills. Another lesson learned by the students was the importance of communication, either between group members or the group and their instructor.

The students also reported learning the importance of incorporating technology into their studies and the value of having different skills regarding instructional videos, including researching, scriptwriting, animation, video editing, and others.

## Conclusion

In conclusion, compared to the traditional lecture format that focuses on memorizing theoretical definitions and structures, instructional videos can be considered an innovative teaching tool and a unique medical education method. Instructional videos allow students to participate more in the learning process. The authors describe its benefits from the perspectives of both teachers and students. However, all presented results are obtained using a student satisfaction survey and a grading rubric. This article presents an active learning method in undergraduate medical education.

## Data Availability

The datasets used and/or analyzed during the current study available from the corresponding author on reasonable request.

## Declarations

### Limitations

The study is a single arm, single center, pre-post study.
